# Evaluation of self-sampling-based cervical cancer screening strategy using HPV Selfy CE-IVD test coupled with home-collection kit: a clinical study in Italy

**DOI:** 10.1186/s40001-023-01263-8

**Published:** 2023-12-11

**Authors:** Giulia Feltri, Giulio Valenti, Erica Isidoro, Jaspreett Kaur, Marianna Treleani, Aurora Bartelloni, Claudia Mauro, Federica Spiga, Giulia Ticich, Michela Di Napoli, Claudia Biagi, Maria Pachetti, Sandro Centonze, Santina Castriciano, Sara Zanchiello, Fabiola Giudici, Daniela Gerin, Fabrizio Zanconati

**Affiliations:** 1 UCO/SC Anatomia e Istologia Patologica, Azienda Sanitaria Universitaria Giuliano Isontina, Cattinara Hospital, Trieste, Italy; 2 Area Science Park, SS 14 - Km 163.5, Trieste, Italy; 3 Cervical Cancer Screening Coordination Unit, Azienda Sanitaria Universitaria Giuliano Isontina, Trieste, Italy; 4 Clinical Research Unit, Azienda Sanitaria Universitaria Giuliano Isontina, Trieste, Italy; 5 Copan Italia S.p.A., via F. Perotti 10, Brescia, Italy; 6 Service de Biostatistique et d’Épidémiologie, Gustave Roussy, Université Paris-Saclay, Villejuif, France; 7 Équipe Labellisée Ligue Contre le Cancer, Oncostat, U1018, Inserm, Université Paris-Saclay, Villejuif, France; 8 Department of Medical Science, University of Trieste, Trieste, Italy

**Keywords:** Human papillomavirus, HPV, Self-sampling, HPV Selfy, VALHUDES, COVID-19

## Abstract

**Background:**

Primary human papillomaviruses (HPV) cervical cancer screening can be strengthened by offering home-collection of biological specimen as a valuable option to increase screening coverage. As recommended by World Health Organization (WHO), screening programs should consider whether the inclusion of HPV self-sampling as a complementary option within their existing screening algorithms could address the gaps in current coverage. However, few HPV screening tests are validated for self-sampling according to international guidelines. This study aimed to test a self-sampling-based screening strategy, complementary to the main screening program based on clinician-collected cervical samples. The study took place in Trieste, Italy, and it aimed to evaluate the feasibility of self-testing at home under an opt-in system during COVID-19 pandemic in order to exploit self-sampling to reduce the screening delay generated by the lockdown.

**Methods:**

500 women, who should have received the screening call in 2020, were asked, via phone call, to participate in the study. To whom agreed, a home-collection kit, including a vaginal dry swab for specimen collection, was sent. The recipients performed the sample self-collection and sent back the swab through traditional mail using a prepaid envelope. Once received by the hospital, the samples were analyzed with HPV Selfy (Ulisse BioMed, Italy), a CE-IVD HPV screening test specifically validated for self-collection. Results were further compared using cobas^®^ 4800 HPV (Roche, Switzerland).

**Results:**

80% women sent back their swab, showing one of the highest return rate obtained in comparable studies. 34 HPV-positive women were followed up and underwent the Pap test, that revealed 8 low squamous intraepithelial lesions (LSIL) cases, later triaged to colposcopy. HPV Selfy was confirmed to be an adequate test for self-sampling-based screening.

**Conclusions:**

This study further confirmed the feasibility of self-test at home screening strategy based on self-sampling with an opt-in system as a support method to enhance cervical cancer screening coverage in Italy. Enrolled women showed a high appreciation for this approach. HPV Selfy test demonstrated to be a valuable assay for cervical cancer screening based on home self-collection.

*Trial registration*: ASUGI Trieste n. 16008/2018 and amendment 02-11/09/2020.

**Supplementary Information:**

The online version contains supplementary material available at 10.1186/s40001-023-01263-8.

## Introduction

Cervical cancer is the fourth most common female cancer worldwide with 604,237 new cases and 341,843 deaths in 2020 [1], of which more than 90% are found in low-income countries due to the lack of cervical screening programs [2, 3]. Indeed, the preventive effect of cervical cancer screening largely depends on the high women participation and coverage. A large number of cervical cancers diagnoses normally arise among under-screened and unscreened women [4–6]. Increasing the screening coverage is essential to improve the effectiveness of primary cervical screening programs [7, 8].

The validation of human papillomavirus virus (HPV) molecular tests on self-collected samples offers the opportunity to adopt this collection method for primary screening purposes with the final aim to increase cervical cancer screening coverage. In particular, by means of self-sampling WHO aims to reach a global target of 70% screening coverage by 2030 [9]. This implementation is allowed by the substantial equivalence, in terms of quality and quantity, of self-collected vaginal samples in comparison with clinician-collected cervical specimens. Indeed, self-samples have been shown to be mostly equivalent [10, 11]; in particular flocked swabs have the peculiarity to collect a higher amount of cellular material compared to cotton swabs [12].

In particular, it has been shown that offering to the screening population the possibility to perform a vaginal self-collection specimen at home significantly increases the response rate compared to traditional screening methods based on clinician-collected biological sampling [13–20].

These aspects have been documented through a plethora of studies, reaching participation rates, intended as the percentage of women who returned back the self-collected sample, ranging from 10 to 64% [14, 21–27].

Surveys evaluating women’s experiences with self-sampling showed that most non-attendees would prefer self-sampling to clinician-based sampling in the next screening round [28–30].

Notwithstanding, in 2020, no assays were clinically validated for primary screening purpose specifically on self-collected samples according to the VALHUDES international guidelines [31].

At the time of the present study, only two CE-IVD assays reported self-sampling in their intended use: (a) HPV Selfy, validated for self-collection since May 2019 on a large population of more than 1,000 women [32, 33]; data of clinical trials performed between 2018 and 2019 were recently published and showed clinical validation of the HPV Selfy assay for primary cervical cancer screening purposes according to Meijer’s guidelines and for self-collection based screening program according to requirements indicated in the VALHUDES protocol [34, 35]; and (b) HPV-Risk, developed by SelfScreen and distributed by QIAGEN with the commercial name of “QIAScreen”; its intended use for self-collection was applied in August 2019 and it was validated for self-collection on a very limited sample size [36, 37]; the assay was validated for primary cervical cancer screening purposes, according to Meijer’s guidelines, but it was not, and it is still not, validated according for cervical cancer primary screening on self-collected samples, according to VALHUDES protocol [31].

However, the introduction of HPV self-test at home based on self-sampling, both as an opt-in system (i.e., the self-sampling device is sent only to the women who requested it) or an opt-out system (i.e., the self-sampling device is sent to all the relevant screening population), could be a valuable and effective strategy to increase primary cervical cancer screening coverage. Indeed, different studies showed how self-sampling can increase the participation in cervical cancer screening both in developed and developing countries [4, 6, 38–42]. Opt-in system appears in general to achieve higher cost-effectiveness, since the home-collection kit is sent only to those women who expressed an explicit consent over the proposed screening participation method [43, 44].

In addition, this could be of particular interest in the post-COVID-19 era, where self-sampling could represent a timely, accessible, safe and cost-effective method to efficiently screen women while keeping social distance [45–50]. Importantly, since 2020, complete lockdown due to pandemic, led to the temporary suspension of HPV screening, forcing to postpone women’s scheduled screening test. Moreover, even after the lockdown release, social distancing measures that in some countries lasted for 3 years forced health departments to restrict access to obstetrician ambulatories to a limited number of women, diminishing then the daily capacity to collect samples. The overall situation created a bottleneck with queue and delay problems in the execution of the screening program; bottleneck management could be facilitated by offering self-sampling as an opt-out screening system in these circumstances [50]. For instance, Netherlands has already adopted home-testing with the self-collection as primary screening method under an opt-out system [16, 51].

This study was started as a first experimental application of HPV self-test at home based on self-sampling offered to screening population in Trieste (Italy) during the interruption of the primary screening due to COVID-19 pandemic. In order to guarantee trustable results, a CE-IVD marked test specifically validated for self-collected samples (HPV Selfy) was selected for this study [32, 33].

## Methods

### Study design

We applied an opt-in system, since according to some studies, it is more cost-effective on average [43], and we also found it economically convenient considering the study local context. In more details, we offered by a phone call to screening attendants the possibility to perform an HPV self-test at home based on self-sampling while waiting for the reactivation of the traditional primary screening program based on clinician-collected samples. To those women who accepted the offer, self-sampling devices were sent to their homes by regular mail; the women collected the biological samples and returned them by regular mail using a prepaid envelope, since this method appeared to achieve significant return rates in comparable studies [52]. From January 2021 to May 2021, trained midwives called by telephone a randomly selected group of women with the last Pap test dating back to 2017 who should have received the new screening call in 2020, aged between 31 and 66, enrolling consecutively 500 women who explicitly agreed to receive the self-collection kit at home.

The women, during the call, received by the midwives an exhaustive explanation of the initiative, with particular reference to the self-sampling process, sample shipment and to the management of the informed consent form included in the kit. Moreover, the women were provided with a customer care telephone contact dedicated to the project for any further need. The demographic and clinical data of each patient enrolled were collected in a proper database with a case-report form that was filled during the enrollment phone call. Pregnant women and women with current diagnosis of uterine, endometrial, vaginal, vulvar or ovarian cancers were not included in the study.

**Fig. 1:**
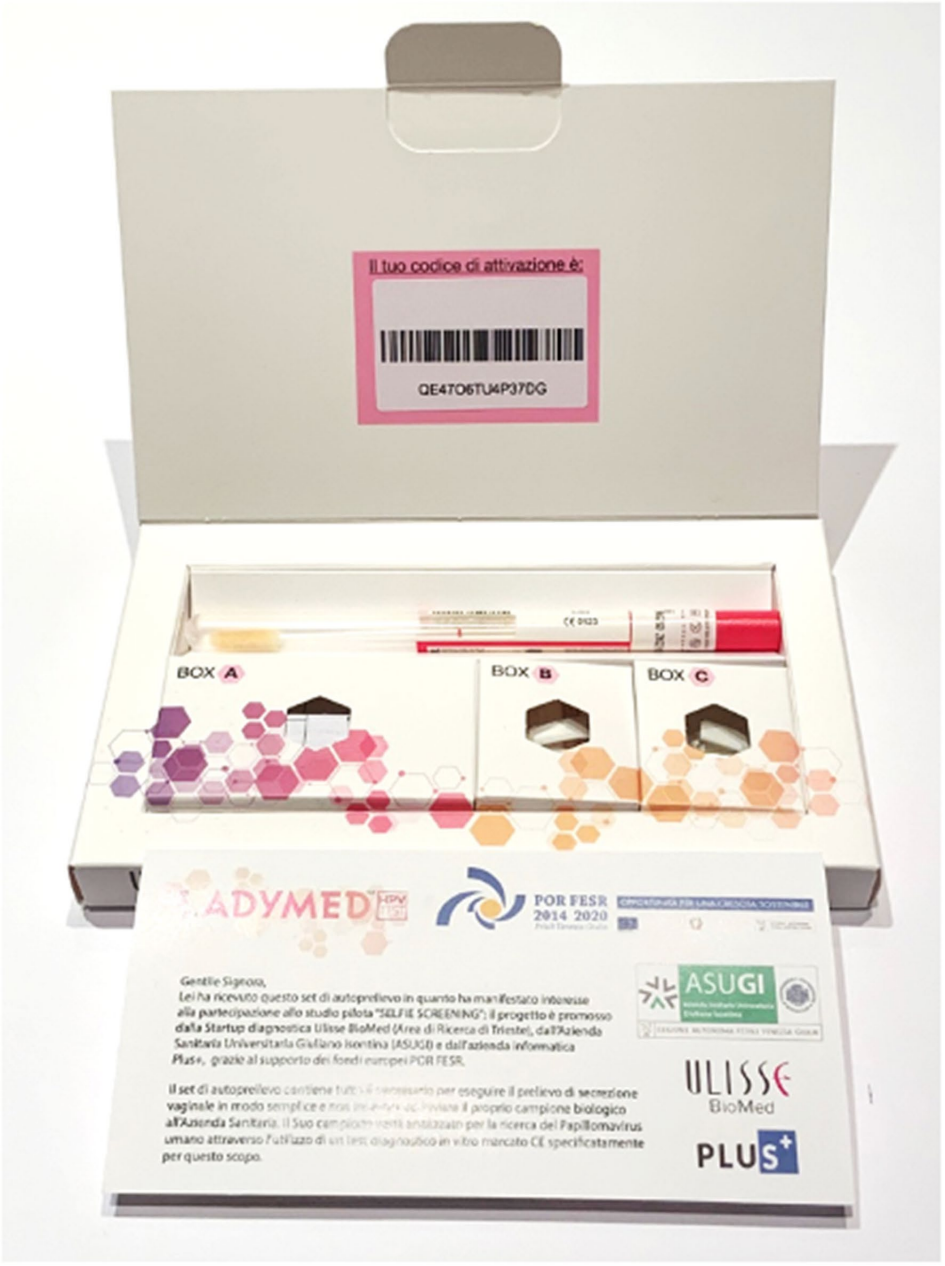
Self-sampling kit manufactured for this study

The self-sampling kits were manufactured by Ulisse BioMed (Fig. 1) and were composed by: a vaginal swab FLOQSwabs^®^ validated for self-collection (Copan, Brescia, Italy) with manufacturer’s instructions of use, general instructions of the self-sampling procedure, the informed consent form to participate to the study, a satisfaction questionnaire containing three questions about their opinion on the study procedure, and a prepaid and pre-addressed envelope to return the sample and the documents by standard postal mailbox to the laboratory of Anatomia e Istologia Patologica, Cattinara Hospital, Trieste, Italy.

If women did not sent samples back, one or two reminders by phone call would have occurred.

### Sampling method

As previously mentioned, for this study, a vaginal swab FLOQSwabs® (Copan, Brescia, Italy) was selected as a self-sampling device. The FLOQSwab® is a dry, sterile, flocked swab contained in a tube used to store the sample after collection to transport to the laboratory. The swab shaft has a red mark to indicate where to hold the swab during collection following a pictorial self-collection guide collection guide. The FLOQSwabs® has been validated for both point of care and home self-collection for the detection of HPV.

### Study procedures

Once the kits were received at their homes, women performed vaginal sample self-collection using sterile dry flocked swab contained in the kit. According to the manufacturer’s instructions, after collection, the swabs are stored dry in their own plastic tube, placed in a plastic bag, packaged and mailed to the hospital together with filled and signed consent documents and a satisfaction questionnaire. Upon receiving the samples envelopes, the hospital laboratory staff registered the arrival date and time and status of the samples.

To quantify the time between the sample self-collection and arrival to the laboratory, the signature date written in the consent form has been taken into consideration as “collection date”.

Vaginal self-collected samples were analyzed by HPV Selfy assay coupled with Ulisse Faster DNA reagent according kit’s instructions for use (Ulisse BioMed S.p.A, Trieste, Italy), that allows to perform direct PCR analysis, thus skipping DNA extraction steps.

To verify the reproducibility over different PCR instruments, the samples were tested twice with HPV Selfy, once with Light Cycler 480 (Roche Molecular Systems, Pleasanton, CA) and once with Quant Studio 5 Real-Time PCR (Thermo Fisher, USA) instruments.

A portion of all the samples were also analyzed by cobas^®^ 4800 HPV (Roche Molecular Systems) and by genotyping test EasyPGX^®^ ready HPV test (Diatech Pharmacogenetics, Jesi, Italy).

Negative test results were communicated by phone. In case of positive HPV test result, the midwives called the positive women and booked a follow-up visit aimed to perform a traditional clinician-collected cervical sampling successively analyzed by cobas^®^ 4800 HPV, used in Trieste for primary cervical cancer screening purposes, and by Pap test. If an abnormal Pap test result was observed during the follow-up, colposcopy was executed.

### HPV testing

HPV Selfy is a CE-IVD full genotyping Real-Time PCR-based HPV screening test capable to detect and perform single genotyping of 14 High-Risk HPV types (16, 18, 31, 33, 35, 39, 45, 51, 52, 56, 58, 59, 66 and 68), thanks to the Ulisse BioMed SAGITTA patented technology, that allows to perform precise genotyping by means of melting curve analysis within the same Real-Time PCR run. The test is at date validated for cervical cancer primary screening, not only on clinician-collected samples, according to Meijer’s international guidelines, but also on self-collected samples, according to the VALHUDES protocol [32, 33].

Prior to perform the analysis with HPV Selfy, the dry swab biological samples were resuspended in 2 ml RNAse- and DNAse-free sterile water, and 80 μl were pre-treated with Ulisse Faster DNA (Ulisse BioMed, Trieste, Italy), a pre-treatment reagent that allows to skip DNA extraction and purification, in order to perform the so-called direct PCR, thus saving time and money. HPV Selfy test includes a human DNA amplification control (Hemoglobin subunit beta) to evaluate biological sample quality, thereby reducing the risk of false-negative results [20]. Analysis with HPV Selfy was performed according to the manufacturer’s protocol, using Quant Studio 5 (Thermo Fisher Scientific; Waltham, Massachusetts, United States) and Light Cycler 480 (Roche Molecular Systems, Pleasanton, CA) Real-Time PCR machines.

Cobas^®^ 4800 HPV is a CE-IVD and FDA-approved fully automated HPV screening test based on Real-Time PCR amplification for the detection of 14 High-Risk HPV types (16, 18, 31, 33, 35, 39, 45, 51, 52, 56, 58, 59, 66 and 68) and partial genotyping of HPV16 and HPV18; the assay is validated for clinician-collected cervical brush samples stored in ThinPrep PreservCyt Solution (Hologic, Marlborough, Massachusetts, USA).

Samples were also analyzed with EasyPGX HPV kit (Diatech Pharmacogenetics, Jesi, Italia), for the detection and genotyping of 14 High-Risk HPV types (16, 18, 31, 33, 35, 39, 45, 51, 52, 56, 58, 59, 66 and 68) through the Real-Time PCR amplification of the oncogenes E6 and E7. For EasyPGX analysis, DNA extraction of the samples was performed using the MagCore HF16 instrument (RBC Bioscience, Zhonge, Taiwan), an automated nucleic acid extractor.

Cervical smear slides were Pap-stained, and histo-technicians interpreted the results following the Bethesda 2001 classification [53].

### Satisfaction questionnaire

Simple questionnaire items assessed participants’ attitudes regarding the offered screening procedure. Attitudes toward the self-collection experience were assessed by evaluating the process with three questions through a satisfaction scale from 1 (very low) to 10 (very high). Questions were adapted from the questionnaire of the VALHUDES study [54]. An open field was left for women willing to express possible comments or concerns on the experience.

### Statistical analysis

The agreement between the collection methods were calculated using concordance and discordance rates and the Cohen’s kappa statistic. The kappa statistic was calculated to determine the level of chance-agreement between two methods with a kappa value of 0 indicating no agreement better than chance, a value of 1 indicating perfect agreement better than chance, and intermediate values of 0.00–0.20, 0.21–0.40, 0.41–0.60, 0.61–0.80 and > 0.81 indicating poor, fair, moderate, substantial and excellent agreement, respectively [55]. The data were presented in percentage, mean and standard deviation as appropriate. *P* values of < 0.05 were taken as statistically significant. All statistical analyses were performed using the Statistical Software R (version 3.5.0, http://www.r-project.org).

## Results

### Swabs reception and study cohort description

Out of 1500 women aged 31–66 contacted by midwives by phone, 500 agreed to participate in the study and were enrolled. Over the 500 swabs sent to the enrolled women, 400 swabs returned to the laboratory (400/500 = 80%) (Table 1A).

**Table 1:** Description of the study cohort

(A) Description of the statistics related to the samples return rate		
	Number|(500)	%
Women who sent the swab	402	80.4
Swabs never arrived to the lab	2	0.4
Total swabs received at the lab	400	80.0
*Swabs arrived without reminder*	319	63.8
*Swabs arrived after 1 reminder*	73	14.6
*Swabs arrived after 2 reminders*	8	1.6
Women who did not send the swab	100	20.0
*Of which women who decided explicitly to not adhere*	25	5.0
*Of which 7 kits were not arrived at home*	7	1.4
Total self-collection kit sent	500	100.0

(B) Age-grouped study cohort			
		Number|(400)	%
**Age**	31–45 years	3	0.8
	46–50 years	98	24.5
	51–55 years	124	31.0
	56–60 years	133	33.3
	61–66 years	42	10.5
	Total	400	100

(C) Previous screening test for the study cohort			
		Number|(400)	%
Previous screening test	Before 2017	28	7.0
	2017	119	29.8
	2018	246	61.5
	2019	1	0.3
	2020	1	0.3
	2021	1	0.3
	Not available	4	1.0
	Total	400	100.0

In particular, 319 swabs were sent back to the hospital without any reminders. The women who did not send the sample were called by the midwives for a first reminder: 73 women sent the sample after this first reminder. A second reminder was done to the remaining women and 8 women sent the sample after the second reminder. 100 women never sent back the swab.

Out of those 100 women, 25 women explicitly communicated their intention to drop-out the clinical study, after receiving the self-collection kit at home. Another 7 women out of these 100 women declared that they never received the self-collection kit at home (7/100 = 7.0%). 2 women declared that they sent their samples back despite they were never received by the laboratory (2/100 = 2.0%).

Additional data regarding the women whose swab was received by the laboratory are described in the Table 1B and C. 396 out of 400 women had a previous screening test result that was negative.

### Laboratory shipping time

Data regarding the lead time of the swabs are presented in Table 2. Most swabs were received within 1 week from the collection date (341/400 = 85.3%); 32 samples were received within 2 weeks and 4 were received within 3 weeks. 3 samples were received between 30 and 48 days from the “collection date”, whereas 20 women (5% of cases) decided to personally restitute their swab at the lab hospital without declaring the collection date.

**Table 2:** Description of the statistics related to the sample arrival time

Sample arrival time to the lab	*n*	%
Within 1 week	341	85.3
Within 2 weeks	32	8.0
Within 3 weeks	4	1.0
More than 1 month (30–48 days)	3	0.8
Women who returned personally the swab at the health district	20	5.0
Total	400	100.0

Most samples arrived within 1 week at the laboratory

### HPV Selfy analysis

We analyzed the 400 returned swabs with Quant Studio 5, following manufacturer’s instructions; 2 samples were invalid due to low or null control gene amplification (2/400 = 0.5%). Out of 398 valid samples, 364 samples were HPV-negative (364/398 = 91.4%), whereas 34 samples were HPV-positive with at least one type of High-Risk HPV (8.5%), being HPV prevalence similar to that reported in previous literature [32]. We found 5 HPV co-infections (double infections) and 29 single infections. Frequency of each genotype is reported in Table 3. Surprisingly we observed that the most frequent HPV type was HPV68 (15.4%, 6 infections), followed by HPV16 (12.8%, 5 infections). The HPV68-positive samples were, for further assurance, confirmed as HPV68-positive by EasyPGX HPV genotyping kit.

**Table 3:** Genotyping performed by HPV Selfy in the study cohort

HPV genotype	*n*	%
HPV 68	6	15.4
HPV 16	5	12.8
HPV 58	4	10.3
HPV 66	4	10.3
HPV 52	4	10.3
HPV 56	4	10.3
HPV 31	3	7.7
HPV 59	3	7.7
HPV 45	2	5.1
HPV 18	2	5.1
HPV 33	1	2.6
HPV 39	1	2.6
HPV 35	0	0.0
HPV 51	0	0.0
Total	39	100.0

HPV68 was the most frequently observed HPV type among 39 infections, followed by HPV16

To evaluate the assay inter-device reproducibility, we performed a second run of the HPV Selfy test on another instrument, the Light Cycler PCR machine: 3 invalid samples were found (3/400 = 0.75%), of which two were the same invalid samples detected by Quant Studio 5. Therefore, 397 samples were considered for the comparison. Agreement between the paired results obtained over the two machines was 99.5% (Cohen’s Kappa 0.97: 95% CI 0.92–1.00—almost perfect agreement), demonstrating high grade of reproducibility of the assay (Additional file 1: Table S1).

### Comparison with other tests

The 400 swabs were also analyzed by cobas^®^ 4800 HPV assay: 4 samples tested with this method were found invalid by Cobas (4/400 = 1%), of which 1 was found invalid also by HPV Selfy performed on Quant Studio 5. cobas^®^ 4800 HPV detected 372 negative cases (372/396 = 93.9%) and 24 HPV-positive cases (24/396 = 6%), 10 fewer than HPV Selfy.

In order to compare HPV Selfy with cobas^®^ 4800 HPV results, we considered 395 samples that were valid for both assays, showing a total agreement of 95.9% (Cohen’s Kappa 0.70: 95% CI 0.56–0.85—substantial agreement) (Table 4). Thus, 16 samples gave discordant results using the two different assays. We decided to re-test those discordant cases with a third CE-IVD genotyping test, the EasyPGX HPV assay. Out of 16 discordant cases, we found 10 cases that were in agreement with HPV Selfy results (10/16 = 62.5%), whereas 6 were in agreement with Cobas (37.5%). These 10 cases, where EasyPGX HPV kit results agreed with HPV Selfy ones, were negative to Cobas. The re-test with EasyPGX determined that these 10 cases were true HPV-positive. As a result of the correction of the Cobas outcome with the EasyPGX re-test, discordant samples went from 16 to 6, implying an adjusted concordance of 98.5% (almost perfect agreement, Kappa Cohen 0.90).

**Table 4:** Comparison between HPV Selfy and cobas^®^ 4800 HPV in the study cohort (*n* = 395)

Cobas^®^ 4800 HPV		HPV Selfy		Total
		HR-HPV negative	HR-HPV positive	
HR-HPV negative	Negative	358	13	371
HR-HPV positive	Positive	3	21	24
	Total	361	34	395

395 self-collected samples were tested with HPV Selfy according to the manufacturer’s protocol, as well as with cobas^®^ 4800 HPV. Total agreement (95.9%) was raised to 98.5% after adjustment with third test CE-IVD test result on the discordant population

### Follow-up of HPV-positive women

34 women tested positive with HPV Selfy were called for a follow-up visit at the health district for a cervical brush sampling by midwives to perform Pap test and to re-test HPV with cobas^®^ 4800 HPV assay with the proper specimen meant to be used by cobas^®^ 4800 HPV according to its intended use.

Out of 34 recalled women, 33 showed up to the health district for the gynecological visit, where a cervical brush sample was collected by the midwives and stored in ThinPrep PreservCyt solution.

These samples were analyzed by Pap test and cobas^®^ 4800 HPV assay.

Out of 33 analyzed samples, 9 samples were HPV-negative with Cobas and 24 were HPV-positive.

Over the same 33 women, we obtained 21 positive women when cobas^®^ 4800 HPV assay was performed on vaginal self-collected samples; and 24 positive women when cobas^®^ 4800 HPV assay was performed on clinician-collected cervical specimen.

Consequently, the number of discordant samples between cobas^®^ 4800 HPV assay and HPV Selfy was reduced from 13 to 10, demonstrating that the assays are more trustable if used on the sample type indicated in their intended use. Regarding the 9 discordant cases (HPV-positive with HPV Selfy on self-collected samples, and HPV-negative with cobas^®^ 4800 HPV on clinician-collected cervical specimen), the clinician-collected cervical specimen were retested with EasyPGX^®^ ready HPV test, according to its intended use, and 6 out of 9 retested samples resulted HPV-positive confirming the result previously obtained with HPV Selfy on self-samples (30/33; adjusted agreement 90.9%).

On the same 33 samples we performed also cytology analysis through Pap test: we found 8 Low-grade Squamous Intraepithelial Lesions (LSIL) cases (LSIL, 8/33 = 24.2% of cases; 8/400 = 2% of samples received), all detected as HPV-positive with HPV Selfy on self-collected samples as well as with cobas^®^ 4800 HPV assay on clinician-collected samples.

However, 2 out of 8 LSIL cases (25%) were negative with cobas^®^ 4800 HPV assay performed on self-collected samples, out of its intended use, thus further confirming the importance to use tests clinically validated on self-collected samples in order to perform screening self-test at home (Table 5).

**Table 5:** Comparison between HPV Selfy and cobas^®^ 4800 HPV in the LSIL subpopulation (*n* = 8)

cobas^®^ 4800 HPV		HPV Selfy		Total
		HR-HPV negative	HR-HPV positive	
HR-HPV negative	Negative	0	2	2
HR-HPV positive	Positive	0	6	6
	Total	0	8	8

HPV Selfy was able to detect all women later diagnosed with LSIL based on their self-collected sample, whereas cobas^®^ 4800 HPV performed on the same sample failed to detect positive 2 of the LSIL diagnosed women (25%)

All the LSIL-positive women were subjected to colposcopy obtained a negative result, thus a 1-year follow-up was scheduled for them.

### Evaluation of women’ satisfaction

**Fig. 2:**
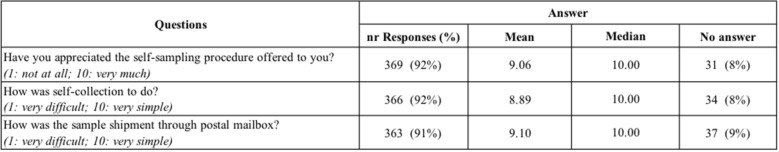
Summary of the questionnaire’ results regarding this study. Women expressed very favorable consent toward self-sampling-based cervical cancer screening approach

Enclosed within the kit, a survey form with three simple questions regarding the screening project based on self-collection was administered to the participants. To each question, the women could provide a satisfaction indicator, ranging from very low (1) to very high (10). Overall women appreciated the project proposal very much, with 9.01 average (Fig. 2). The answers to each question are summarized into Fig. 2**.**

## Discussion

HPV testing on home self-collected vaginal specimens is an effective primary cervical cancer screening method, valuable both to recruit non-responders and to screen the entire relevant screening population. HPV screening based on self-collection of dry swabs indeed could be advantageous since (i) the self-collection-based screening does not require specialized personnel for the collection step, thus simplifying the organization and lowering the costs; (ii) it could reach women who do access the gynecological visit by cultural, religious, psychological or other social barriers; (iii) it ensures to achieve equivalent performance compared to cervical collection, if the HPV test method is specifically validated for this purpose. In addition, recent studies showed that more extensive self-collection in the anogenital area (especially in the anal zone) could even allow detection of HPV reservoirs that could have an impact on cervical and not-cervical HPV-cancer development [56–58].

We showed that HPV Selfy, a test validated for primary screening purposes according to Meijer’s guidelines and specifically validated also on self-collected samples according to VALHUDES protocol [32, 33], has an overall higher diagnostic performance (intended as higher sensitivity and specificity) than off-label use of assays that have not self-collection in their intended us.

Moreover, HPV Selfy generated a lower number of inadequate samples (i.e., 50%: 0.5% invalid samples for HPV Selfy vs 1% invalid samples for cobas^®^ 4800 HPV assay), possibly because HPV Selfy assay was specifically optimized for self-collected samples and had this claim in its intended use, while Cobas had not [32, 33, 59]. By means of HPV Selfy, we were able to correctly identify and follow-up 8 LSIL cases corresponding to 2% of the 400 enrolled women who returned the swabs. As the study showed, the use for self-testing at home of a test not validated on self-collected samples would lead to miss 2 LSIL-positive cases (corresponding to 25% of the total LSIS cases).

These results further encourage the effectiveness and feasibility of a “self-collection at home” based cervical cancer screening program.

However, implementation of self-sampling as a primary cervical cancer screening collection method needs to consider the return rate as an essential parameter to evaluate cost-effectiveness of the procedure.

Indeed, if on one hand self-sampling would reduce the costs of cervical screening as it obviates the need of clinician-performed cervical specimen collection at the health districts, on the other side, however, self-sampling studies usually show modest return rate of the swabs, meaning that under an opt-out system only a modest percentage of the swabs could be sent back to the lab. Thus, healthcare policy makers should take into consideration, under an opt-out system, the cost of the deployment of a high number of home-collection kits with a possible low participation rate. Notwithstanding, countries with the most developed screening program, such as the Netherlands or Australia, are already adopting self-testing at home under an opt-out system as the main primary cervical cancer screening sample-collection method [16, 51].

A recent review compared the different invitation strategies for self-sampling and results, ranging from very scarce (6.4% of an opt-out study in Sweden) up to very high return rate (39.0% of opt-in study in Sweden, as well as 39.1% of opt-out study in Finland), show that there is not a significant difference among invitation methods, although it has to be underlined that the studies were different in the setup (invitation modalities; delivery and restitution modalities..), in the sampling devices (cervical brushes, vaginal swabs, vaginal veils, urine collectors, etc.), in the sociodemographic and economical background of the investigated population and, most importantly, in the communication strategy (type of information and instructions for use provided with the self-collection kit, local involvement and communication, digital communication, etc.) [60, 61]. A recent meta-analysis that analyzed 33 clinical trials concluded that opt-in strategies were less effective than send-to-all strategies [62], although many other studies did not find a significant difference between the strategies in terms of return rate [for instance 43 and 63]. It is evident that a clear-cut consensus has not yet built up in the scientific literature, the final decision on which strategy to undertake on a certain population should be based on appropriate cost-effectiveness studies that consider the local context and resources of the studied population. With the present study based on an adapted opt-in system, instead, we showed that it is possible to achieve high cost-effectiveness as well as a method with an extremely high rate of success, intended as return rate (80%, the highest described in the literature at date, that doubles the previous highest return rates measured—39% described in an opt-out study in Finland [20] and 39.1% described in a opt-in study in Sweden [25]). The adopted opt-in system allowed to save important resources and to send the home-collection kit only to those women who expressed an explicit consent over the proposed screening participation method.

A possible bias of this result could be due to the fact that we selected women who had at least a screening test in their life (so we focused on a more responsive population by excluding the non-attendee’s population), however, it has been previously shown that also screening attendees, expressed their preference for self-sampling compared to clinician-based sampling [32], alike most non-responders. Thus, we could partially explain the observed high return rate because of the implemented opt-in system based on an individual pre-screening phone call, that allowed us to deliver the kits only to the women who expressed their willingness to participate to a self-sampling-based screening, thus, making the process more efficient and targeted.

Other possible factors leading to this result include the fact that we provided a user-friendly home-collection kit, with clear instructions for use, and the entire study was done with a simple returning method (i.e., shipment via the postal mailboxes, widely diffused over the territory and accessible with the maximum privacy and any time, using a prepaid and pre-addressed envelope). Finally, we also provided a dedicated customer care telephone contact to the women, to provide them any required assistance.

Next step will be to enlarge and further validate the established opt-in protocol also to non-responders’ population, aiming to increase the screening participation even among those hard-to-reach women.

## Conclusions

In this clinical study performed on 500 enrolled women, we verified that HPV Selfy test is a suitable assay for primary cervical cancer screening programs based on self-collection performed at home. Thus, we confirmed that the clinical performance of HPV Selfy executed on self-collected vaginal samples is higher than the performance, on the same self-collected samples, of assays that do not have self-collection in their intended use, therefore, confirming that the HPV Selfy test is suitable to be used in primary cervical cancer screening programs based on self-sampling.

We also demonstrated that the opt-in system we set up was effective and provided a very high return rate, confirming that this system could be used to increase non-responders’ participation rate to screening, and that by the time could be used as the main primary cervical cancer screening collection method.

### Supplementary Information

**Additional file 1: Table S1.** Intralaboratory reproducibility of HPV Selfy using two different Real Time PCR machines. HPV Selfy assay was performed twice on 397 samples using two different Real Time PCR instruments: Quant Studio 5, indicated by the manufacturer’s protocol, and Light Cycler. Overall concordance observed was 99.5% (kappa value of 0.97, almost perfect agreement).

## Data Availability

All data generated or analyzed during this study are included in this published article [and its Additional file 1].

## Abbreviations

HPV: Human papillomavirus
PCR: Polymerase chain reaction
ASUGI: Azienda Sanitaria Universitaria Giuliano Isontina
HR-HPV: High-risk human papillomavirus
OR: Odd ratios
CIN: Cervical intraepithelial neoplasia
CE-IVD: CE-marked in-vitro diagnostics
CI: Confidence interval
WHO: World Health Organization
LSIL: Low-grade squamous intraepithelial lesions

## References

[1] Sung H, Ferlay J, Siegel RL, Laversanne M, Soerjomataram I, Jemal A, Bray F (2021). Global cancer statistics 2020: GLOBOCAN estimates of incidence and mortality worldwide for 36 cancers in 185 countries. CA Cancer J Clin.

[2] Sankaranarayanan R, Thara S, Esmy PO, Basu P (2008). Cervical cancer: Screening and therapeutic perspectives. Med Princ Pract.

[3] Crofts V, Flahault E, Tebeu PM (2015). Education efforts may contribute to wider acceptance of human papillomavirus self-sampling. Int J Womens Health.

[4] Gök M, Heideman DAM, Van Kemenade FJ (2012). Offering self-sampling for human papillomavirus testing to non-attendees of the cervical screening programme: characteristics of the responders. Eur J Cancer.

[5] Tranberg M, Bech BH, Blaakær J, Jensen JS, Svanholm H, Andersen B (2018). Preventing cervical cancer using HPV self-sampling: direct mailing of test-kits increases screening participation more than timely opt-in procedures - a randomized controlled trial. BMC Cancer.

[6] Haguenoer K, Sengchanh S, Gaudy-Graffin C (2014). Vaginal self-sampling is a cost-effective way to increase participation in a cervical cancer screening programme: a randomised trial. Br J Cancer.

[7] Crosbie EJ, Einstein MH, Franceschi S, Kitchener HC (2013). Human papillomavirus and cervical cancer. Lancet.

[8] ICO HPV Information Centre. Human Papillomavirus and Related Diseases Report. HPV Inf Cent. 2017;60.

[9] https://apps.who.int/iris/rest/bitstreams/1280112/retrieve. Accessed 04 Sept 2022.

[10] Sechi I, Elvezia CC, Martinelli M, Muresu N, Castriciano S, Sotgiu G, Piana A (2022). Comparison of different self-sampling devices for molecular detection of human papillomavirus (HPV) and other sexually transmitted infections (STIs): a pilot study. Healthcare.

[11] Saville M, Hawkes D, Keung MHT, Ip ELO, Silvers J, Sultana F, Malloy MJ, Velentzis LS, Canfel K, Wrede CD, Brotherton JML (2020). Analytical performance of HPV assays on vaginal self-collected vs practitioner-collected cervical samples: the SCoPE study. J Clin Virol.

[12] Viviano M, Willame A, Cohen M, Benski AC, Catarino R, Wuillemin C, Tran PL, Petignat P, Vassilakos P (2018). A comparison of cotton and flocked swabs for vaginal self-sample collection. Int J Womens Health.

[13] Verdoodt F, Jentschke M, Hillemanns P, Racey CS, Snijders PJF, Arbyn M (2015). Reaching women who do not participate in the regular cervical cancer screening programme by offering self-sampling kits: a systematic review and meta-analysis of randomised trials. Eur J Cancer.

[14] Szarewski A, Cadman L, Mesher D (2011). HPV self-sampling as an alternative strategy in non-attenders for cervical screening- a randomised controlled trial. Br J Cancer.

[15] Snijders PJF, Verhoef VMJ, Arbyn M (2013). High-risk HPV testing on self-sampled versus clinician-collected specimens: a review on the clinical accuracy and impact on population attendance in cervical cancer screening. Int J Cancer.

[16] Inturrisi F, Aitken CA, Melchers WJG, van den Brule AJC, Molijn A, Hinrichs JWJ, Niesters HGM, Siebers AG, Schuurman R, Heideman DAM, de Kok IMCM, Bekkers RLM, van Kemenade FJ, Berkhof J (2021). Clinical performance of high-risk HPV testing on self-samples versus clinician samples in routine primary HPV screening in the Netherlands: an observational study. Lancet Reg Health Eur.

[17] Jalili F, O'Conaill C, Templeton K, Lotocki R, Fischer G, Manning L, Cormier K, Decker K (2019). Assessing the impact of mailing self-sampling kits for human papillomavirus testing to unscreened non-responder women in Manitoba. Curr Oncol.

[18] Sormani J, Kenfack B, Wisniak A, Moukam Datchoua A, Lemoupa Makajio S, Schmidt NC, Vassilakos P, Petignat P (2021). Exploring factors associated with patients who prefer clinician-sampling to HPV self-sampling: a study conducted in a low-resource setting. Int J Environ Res Public Health.

[19] Cadman L, Reuter C, Jitlal M, Kleeman M, Austin J, Hollingworth T, Parberry AL, Ashdown-Barr L, Patel D, Nedjai B, Lorincz AT, Cuzick J (2021). A randomized comparison of different vaginal self-sampling devices and urine for human papillomavirus testing-predictors 5.1. Cancer Epidemiol Biomarkers Prev.

[20] Gyllensten U, Sanner K, Gustavsson I, Lindell M, Wikström I, Wilander E (2011). Short-time repeat high-risk HPV testing by self-sampling for screening of cervical cancer. Br J Cancer.

[21] Bais AG, vanKemenade FJ, Berkhof J, Verheijen RH, Snijders PJ, Voorhorst F (2007). Human papillomavirus testing on self-sampled cervicovaginal brushes: an effective alternative to protect nonresponders in cervical screening programs. Int J Cancer.

[22] Gok M, Heideman DA, van Kemenade FJ (2010). HPV testing on self collected cervicovaginal lavage specimens as screening method for women who do not attend cervical screening: cohort study. BMJ.

[23] Gok M, van Kemenade FJ, Heideman DA (2012). Experience with high-risk human papillomavirus testing on vaginal brush-based self-samples of non-attendees of the cervical screening program. Int J Cancer.

[24] Bosgraaf RP, Mast PP, Struik-van der Zanden PH, Bulten MLF, Bekkers RL (2013). Overtreatment in a see-and-treat approach to cervical intraepithelial lesions. Obstet Gynecol.

[25] Sanner K, Wikstrom I, Strand A, Lindell M, Wilander E (2009). Self-sampling of the vaginal fluid at home combined with high-risk HPV testing. Br J Cancer.

[26] Smith JS, Des Marais AC, Deal AM (2018). Mailed human papillomavirus self-collection with Papanicolaou test referral for infrequently screened women in the United States. Sex Transm Dis.

[27] Ernstson A, Urdell A, Forslund O, Borgfeldt C (2020). Cervical cancer prevention among long-term screening non-attendees by vaginal self-collected samples for hr-HPV mRNA detection. Infect Agent Cancer.

[28] Huynh J, Howard M, Lytwyn A (2010). Self-collection for vaginal human papillomavirus testing. J Low Genit Tract Dis.

[29] Bosgraaf RP, Verhoef VM, Massuger LF, Siebers AG, Bulten J, de Kuyper-de Ridder GM (2015). Comparative performance of novel self-sampling methods in detecting high-risk human papillomavirus in 30,130 women not attending cervical screening. Int J Cancer.

[30] Nelson EJ, Maynard BR, Loux T, Fatla J, Gordon R, Arnold LD (2017). The ac- ceptability of self-sampled screening for HPV DNA: a systematic review and meta-analysis. Sex Transm Infect.

[31] Peeters E, Benoy I, Vanden Broeck D, Bogers J, De Sutter P, Donders G, Tjalma W, Weyers S, Cuschieri K, Poljak M, Bonde J, Cocuzza C, Zhao FH, Van Keer S, Vorsters A (2018). VALHUDES: a protocol for validation of human papillomavirus assays and collection devices for HPV testing on self-samples and urine samples. J Clin Virol.

[32] Avian A, Clemente N, Mauro E, Isidoro E, Di Napoli M, Dudine S, Del Fabro A, Morini S, Perin T, Giudici F, Cammisuli T, Foschi N, Mocenigo M, Montrone M, Modena C, Polenghi M, Puzzi L, Tomaic V, Valenti G, Sola R, Zanolla S, Vogrig E, Riva E, Angeletti S, Ciccozzi M, Castriciano S, Pachetti M, Petti M, Centonze S, Gerin D, Banks L, Marini B, Canzonieri V, Sopracordevole F, Zanconati F, Ippodrino R (2022). Clinical validation of full HR-HPV genotyping HPV Selfy assay according to the international guidelines for HPV test requirements for cervical cancer screening on clinician-collected and self-collected samples. J Transl Med.

[33] Corrigendum to Avian A, Clemente N, Mauro E, Isidoro E, Di Napoli M, Dudine S, Del Fabro A, Morini S, Perin T, Giudici F, Cammisuli T, Foschi N, Mocenigo M, Montrone M, Modena C, Polenghi M, Puzzi L, Tomaic V, Valenti G, Sola R, Zanolla S, Vogrig E, Riva E, Angeletti S, Ciccozzi M, Castriciano S, Pachetti M, Petti M, Centonze S, Gerin D, Banks L, Marini B, Canzonieri V, Sopracordevole F, Zanconati F, Ippodrino R. Correction: clinical validation of full HR-HPV genotyping HPV Selfy assay according to the international guidelines for HPV test requirements for cervical cancer screening on clinician-collected and self-collected samples. J Transl Med. 2023;21(1):49. 10.1186/s12967-023-03882-5. Erratum for: J Transl Med. 2022;20(1):231. 10.1186/s12967-022-03383-x PMC9115952 35581584

[34] Meijer CJ, Berkhof J, Castle PE, Hesselink AT, Franco EL, Ronco G, Arbyn M, Bosch FX, Cuzick J, Dillner J, Heideman DA, Snijders PJ (2009). Guidelines for human papillomavirus DNA test requirements for primary cervical cancer screening in women 30 years and older. Int J Cancer.

[35] Report n.7 from Gruppo Italiano Screening del Cervicocarcinoma (GISCI) “Test validati per lo screening del cervicocarcinoma”. https://gisci.it/documenti/documenti_gisci/Rapporto_N7_Test_HPV_Validati.pdf. Accessed 04 Sept 2022.

[36] https://herqiagen.com/qiascreen/wp-content/uploads/sites/4/2021/10/HB-2579-003_1117669_R2_QIAScreen_PCR_CE_0819_EMEA.pdf. Accessed 04 Sept 2022.

[37] Hesselink AT, Berkhof J, van der Salm ML, van Splunter AP, Geelen TH, van Kemenade FJ, Bleeker MG, Heideman DA (2014). Clinical validation of the HPV-risk assay, a novel real-time PCR assay for detection of high-risk human papillomavirus DNA by targeting the E7 region. J Clin Microbiol.

[38] Del Mistro A, Frayle H, Ferro A, Fantin G, Altobelli E, Giorgi RP (2017). Efficacy of self-sampling in promoting participation to cervical cancer screening also in subsequent round. Prev Med Rep.

[39] Giorgi Rossi P, Fortunato C, Barbarino P (2015). Self-sampling to increase participation in cervical cancer screening: An RCT comparing home mailing, distribution in pharmacies, and recall letter. Br J Cancer.

[40] Sultana F, English DR, Simpson JA (2016). Home-based HPV self-sampling improves participation by never-screened and under-screened women: results from a large randomized trial (iPap) in Australia. Int J Cancer.

[41] Broberg G, Jonasson JM, Ellis J (2013). Increasing participation in cervical cancer screening: telephone contact with long-term non-attendees in Sweden. Results from RACOMIP, a randomized controlled trial. Int J Cancer.

[42] Vahabi M, Lofters A (2018). HPV self-sampling: a promising approach to reduce cervical cancer screening disparities in Canada. Curr Oncol.

[43] Knauss T, Hansen BT, Pedersen K, Aasbø G, Kunst N, Burger EA (2023). The cost-effectiveness of opt-in and send-to-all HPV self-sampling among long-term non-attenders to cervical cancer screening in Norway: the Equalscreen randomized controlled trial. Gynecol Oncol.

[44] Malone C, Barnabas RV, Buist DSM, Tiro JA, Winer RL (2020). Cost-effectiveness studies of HPV self-sampling: a systematic review. Prev Med.

[45] Lim AWW (2021). Will COVID-19 be the tipping point for primary HPV self-sampling?. Cancer Epidemiol Biomarkers Prev.

[46] Ajenifuja KO, Belinson J, Goldstein A, Desai KT, de Sanjose S, Schiffman M (2020). Designing low-cost, accurate cervical screening strategies that take into account COVID-19: a role for self-sampled HPV typing. Infect Agents Cancer.

[47] Miller MJ, Xu L, Qin J (2021). Impact of COVID-19 on cervical cancer screening rates among women aged 21–65 years in a large integrated health care system—Southern California, January 1–September 30, 2019, and January 1–September 30, 2020. MMWR Morb Mortal Wkly Rep.

[48] EHRN. Delayed Cancer Screenings. https://ehrn.org/articles/delays-in-preventive-cancer-screenings-during-covid-19-pandemic/. Accessed 04 Sept 2022.

[49] Castanon A, Rebolj M, Burger EA, de Kok IMCM, Smith MA, Hanley SJB, Carozzi FM, Peacock S, O'Mahony JF (2021). Cervical screening during the COVID-19 pandemic: optimising recovery strategies. Lancet Public Health.

[50] Lozar T, Nagvekar R, Rohrer C, Dube Mandishora RS, Ivanus U, Fitzpatrick MB (2021). Cervical cancer screening postpandemic: self-sampling opportunities to accelerate the elimination of cervical cancer. Int J Womens Health.

[51] Arbyn M, Latsuzbaia A, Castle PE, Sahasrabuddhe VV, Broeck DV (2022). HPV testing of self-samples: Influence of collection and sample handling procedures on clinical accuracy to detect cervical precancer. Lancet Reg Health Eur.

[52] Ivanus U, Jerman T, Fokter AR, Takac I, Prevodnik VK, Marcec M, Gajsek US, Pakiz M, Koren J, Celik SH, Kramberger KG, Klopcic U, Kavalar R, Zatler SS, Kuzmanov BG, Florjancic M, Nolde N, Novakovic S, Poljak M, Zakelj MP (2018). Randomised trial of HPV self-sampling among non-attenders in the Slovenian cervical screening programme ZORA: comparing three different screening approaches. Radiol Oncol.

[53] Solomon D, Davey D, Kurman R, Moriarty A, O’Connor D, Prey M (2002). The 2001 Bethesda system terminology for reporting results of cervical cytokogy. JAMA.

[54] De Pauw H, Donders G, Weyers S, De Sutter P, Doyen J, Tjalma WAA, Vanden Broeck D, Peeters E, Van Keer S, Vorsters A, Arbyn M (2021). Cervical cancer screening using HPV tests on self-samples: attitudes and preferences of women participating in the VALHUDES study. Arch Public Health.

[55] Hallgren KA (2012). Computing inter-rater reliability for observational data: an overview and tutorial. Tutor Quant Methods Psychol.

[56] Herzum A, Ciccarese G, Drago F, Pastorino A, Dezzana M, Mavilia MG, Sola S, Copello F, Parodi A (2022). Cervical, oral and anal Human papillomavirus infection in women attending the Dermatology Unit of a regional reference hospital in Genoa, Italy: a prevalence study. J Prev Med Hyg.

[57] Ring LL, Thomsen LT, Haedersdal M, Sørensen SS, Bonde JH, Lok TT, Larsen HK, Kjaer SK (2023). Prevalence of cervical human papillomavirus and the risk of anal co-infection in kidney transplant recipients: results from a Danish clinical study. Transpl Infect Dis.

[58] Ciccarese G, Herzum A, Rebora A, Drago F (2017). Prevalence of genital, oral, and anal HPV infection among STI patients in Italy. J Med Virol.

[59] https://pim-eservices.roche.com/eLD/api/downloads/3cfba842-99ed-ec11-1791-005056a71a5d?countryIsoCode=it. Accessed 04 Sept 2022.

[60] Bonde J, Ejegod D. Self-sampling to reach non-participating women. 58. 2018. www.HPVWorld.com. https://www.hpvworld.com/media/29/media_section/6/0/960/hpvworld-058.pdf. Accessed 04 Sept 2022.

[61] Daponte N, Valasoulis G, Michail G, Magaliou I, Daponte A-I, Garas A, Grivea I, Bogdanos DP, Daponte A (2023). HPV-based self-sampling in cervical cancer screening: an updated review of the current evidence in the literature. Cancers.

[62] Costa S, Verberckmoes B, Castle PE, Arbyn M (2023). Offering HPV self-sampling kits: an updated meta-analysis of the effectiveness of strategies to increase participation in cervical cancer screening. Br J Cancer.

[63] Di Gennaro G, Licata F, Trovato A, Bianco A (2022). Does self-sampling for human papilloma virus testing have the potential to increase cervical cancer screening? An updated meta-analysis of observational studies and randomized clinical trials. Front Public Health.

