# Supplementary material for: Evaluation of self-sampling-based cervical cancer screening strategy using HPV Selfy CE-IVD test coupled with home-collection kit: a clinical study in Italy
Source: Eur J Med Res. 2023 Dec 11;28:582. doi: 10.1186/s40001-023-01263-8 (PMC10712215; doi:10.1186/s40001-023-01263-8)
Supplement: Supplementary file 1 — Additional file 1: Table S1. Intralaboratory reproducibility of HPV Selfy using two different Real Time PCR machines. HPV Selfy assay was performed twice on 397 samples using two different Real Time PCR instruments: Quant Studio 5, indicated by the manufacturer’s protocol, and Light Cycler. Overall concordance observed was 99.5% (kappa value of 0.97, almost perfect agreement). [file 40001_2023_1263_MOESM1_ESM.docx]

**Supplementary Information**

**Table S1. Intralaboratory reproducibility of HPV Selfy using two different Real Time PCR machines.** HPV Selfy assay was performed twice on 397 samples using two different Real Time PCR instruments: Quant Studio 5, indicated by the manufacturer’s protocol, and Light Cycler. Overall concordance observed was 99.5% (kappa value of 0.97, almost perfect agreement).
